# Attention Network Test fMRI data for participants with Parkinson’s disease and healthy elderly

**DOI:** 10.12688/f1000research.19288.1

**Published:** 2019-06-04

**Authors:** Trevor K. M. Day, Tara M. Madhyastha, Mary K. Askren, Peter Boord, Thomas J. Montine, Thomas J. Grabowski

**Affiliations:** 1 Department of Radiology, University of Washington, Seattle, WA, 98195, USA; 2 Department of Psychology, University of Washington, Seattle, WA, 98195, USA; 3 Department of Pathology, Stanford University, Stanford, CA, 94305, USA; 4 Department of Neurology, University of Washington, Seattle, WA, 98195, USA

**Keywords:** Attention Network Task, ANT, fMRI, Parkinson’s disease, attention

## Abstract

Here, we present unprocessed and preprocessed Attention Network Test data from 25 adults with Parkinson’s disease and 21 healthy adults, along with the associated defaced structural scans. The preprocessed data has been processed with a provided Analysis of Functional NeuroImages *afni_proc.py* script and includes structural scans that were skull-stripped before defacing. All acquired demographic and neuropsychological data are included.

## Introduction

Attention dysfunction is a common symptom of Parkinson’s disease (PD) and has a significant impact on quality of life. Approximately half of all people with PD suffer from attention and/or memory symptoms ( Barone *et al*., 2009).

The data included here is a subset of data from a study ( Cholerton *et al*., 2013) that used the Attention Network Test (ANT) ( Fan *et al*., 2005) to measure three aspects of attention: alerting (achieving and maintaining an alert state), orienting (selecting the spatial location of sensory input), and executive control (resolving conflict). We acquired structural and functional MRI images at two occasions in participants with and without PD, with six randomly ordered repetitions of the ANT task (labeled 1–6) at each occasion. Each numbered run represents the same stimulus list between subjects, although the six runs were presented to each subject in a different order.

Data described in this paper have previously been analyzed in Boord *et al*. (2017) and Madhyastha *et al*. (2015), wherein the runs were labeled A-F rather than 1–6.

## Materials and methods

### Ethical statement

Procedures were approved by the University of Washington Institutional Review Board (#41304) and subjects provided written informed consent.

### Participants

The sample of subjects includes 25 participants with PD and 21 healthy controls (HC) who participated in two scanning sessions, which were one to three weeks apart. PD participants were recruited from a larger parent study where they underwent extensive clinical examination and neuropsychological assessment ( Cholerton *et al*., 2013).

Demographic information is provided in Table 1. PD and HC participants did not differ on age (t(40) = 1; p = 0.2) or years of education (t(40) = 0.6, p = 0.6), but did differ on the Movement Disorder Society-sponsored revision of the Unified Parkinson’s Disease Rating Scale (MDS-UPDRS; Goetz *et al*., 2007) part III (t(30) = 10; p < .001). Participants also underwent a battery of neuropsychological tests ( Cholerton *et al*., 2013). Neuropsychological test results are provided in Table 2. PD and HC participants did not differ on any of the cognitive tests that were administered to both groups. HC participants obtained only a subset of the measurements.

**Table 1.** Demographics of sample. Participants with PD and healthy controls did not differ on age, UPDRS III, or years of education.

	Parkinson Disease	Healthy Controls
N	25	21
Age (years)	66.1 (10.0)	62.1 (9.9)
Sex (number of males)	18	9
Hoehn & Yahr	2.0 (0.3)	
UPDRS I	10.0 (5.7)	
UPDRS II	8.8 (5.3)	
UDPRS III	23.6 (8.7)	0.8 (1.4)
UPDRS IV	2.0 (3.7)	
Years since disease onset	8.4 (4.8)	
Education (years)	16.2 (2.1)	15.9 (2.4)
Handedness (# right)	21	20

**Table 2.** Summary statistics for cognitive variables. Controls are included where they were administered the exam.

	Parkinson Disease	Healthy Controls
BVRT total correct (delayed)	1 (1.35)	
Copy of Cube	0.78 (0.42)	0.81 (0.4)
Backward digit span	7.46 (2.45)	
Forward digit span	9.5 (1.59)	
Digit span total score	17.08 (3.49)	
Clock drawing (total)	12.44 (1.34)	12.62 (0.86)
Stroop total correct	189.26 (24.99)	
JLO total correct	12.69 (1.89)	
Letter number sequencing total	10.15 (2.51)	
Logical Memory Test (total delay story units recalled)	9.75 (4.55)	
Logical Memory Test (total immediate story units recalled)	11.92 (3.78)	
Logical Memory Test (recognition total score for Story A)	11.69 (2.06)	
Mattis Dementia Rating Scale	138.81 (3.76)	
MMSE score	28.69 (1.19)	
MoCA score	26.44 (2.06)	27.29 (1.95)
Shipley-2 Vocabulary	34.85 (3.86)	
Tower of London total correct	4.7 (3.15)	
Tower of London total time	349.25 (163.78)	
Trail Making Test A-B (s)	-44.69 (29.32)	
Trail Making Test A (s)	29.73 (10.53)	
Trail Making Test B (s)	74.42 (31.53)	
WAIS Digit Symbol score	47.73 (7.94)	

**Abbreviations:** Boston Visual Retention Test (BVRT); Judgment of Line Orientation (JLO); Mini-Mental State Examination (MMSE); Montreal Cognitive Assessment (MoCA); Weschler Adult Intelligence Scale (WAIS)

One subject (RC4206) had an acquisition error during their second session structural scan. Correspondingly, their structural scan from their first session has been copied for their second session to create a valid Brain Imaging Data Structure (BIDS) directory.

### MRI acquisition

At each of the two sessions, we acquired six repetitions of the task and T1-weighted structural images from each subject. Data were acquired using a Philips 3.0T X-Series Achieva MR System (Philips Medical Systems, software version R2.6.3) with a 32-channel SENSE head coil. Each session included functional and structural scans. For task scans, whole-brain axial echo-planar images (43 sequential ascending slices, 3 mm isotropic voxels, field of view = 240 x 240 x 129 mm, repetition time = 2400 ms, echo time = 25 ms, flip angle = 79°, SENSE acceleration factor = 2) were collected parallel to the AC-PC line. Each functional scan was 149 volumes (5.96 min). A sagittal T1-weighted 3D MPRAGE (176 slices, matrix size = 256 x 256, inversion time = 1100 ms, turbo-field echo factor = 225, repetition time = 7.46 ms, echo time = 3.49 ms, flip angle = 7°, shot interval = 2530 ms) with 1 mm isotropic voxels was also acquired for registration and tissue analyses.

In total, 45 subjects completed all six task scans in both sessions. One subject did not complete the second session; and one subject is missing task data for the first four task scans (out of six) at the second session.

Most scans were available in the Digital Imaging and Communications in Medicine (DICOM) file format; and were converted to the Neuroimaging Informatics Technology Initiative (NIfTI) file format using the Analysis of Functional NeuroImages (AFNI) program *dcm2niix_afni*. Subjects with missing DICOMs had Philips format PAR/RECs available and were also converted to NIfTI format using AFNI *dcm2niix_afni* ( Day *et al*., 2019).

### ANT

We used the ANT ( Fan *et al*., 2005; Fan *et al*., 2002), which combines cues and targets within a single reaction time task to measure the efficiency of the alerting, orienting, and executive attention networks. Each session included six separate task runs. Each run included two buffer trials followed by 36 reaction time trials (a total of 432 trials per subject).

A full description of the ANT can be found in Fan *et al*. (2005). Briefly, in the ANT, subjects are asked to determine the direction of an arrow (left or right); which is flanked by four other arrows. These flanker arrows either point the same direction as the probe arrow (“congruent”) or the opposite direction (“incongruent”). The row of arrows appears either above or below the center of the screen, and prior to displaying the arrows, the participants are presented with a) no cue; b) a spatial cue that reflects where the arrows will appear; or c) a center cue. A fixation cross appeared throughout the trial.

### fMRI preprocessing

fMRI data were preprocessed using AFNI ( Cox, 1996), version AFNI_17.3.00 (Oct. 12, 2017). Processing steps were generated with *afni_proc.py* (version 5.18, Sept. 12, 2017), treating each repetition of the ANT task as a single scan (i.e. no concatenation).

***afni_proc.py call***

First four parameters are set on a per-subject basis and represented here with asterisks (*).


afni_proc.py \
      -subj_id			*						\
      -dsets                    *                                       \
      -outdir			*						\
      -script			*						\
      -copy_anat                T1.nii.gz					\
      -blocks despike tshift align tlrc volreg blur mask regress 	\
      -align_opts_aea		-cost	lpc+ZZ				\
      -tlrc_base		MNI152_T1_2009c+tlrc			     	\
      -tlrc_NL_warp							\
      -volreg_warp_dxyz         2                                       \
      -volreg_align_e2a							\
      -volreg_tlrc_warp							\
      -volreg_align_to		MIN_OUTLIER				\
      -regress_anaticor							\
      -regress_est_blur_epits						\
      -regress_est_blur_errts				



We used the following blocks: despike, tshift (default), align, tlrc, volreg (default), blur (default), regress (default). Frames were despiked and slice-timing corrected (tshift). During the align stage, we aligned the functional to the structural using the *lpc+ZZ* cost function. Following structural alignment, we aligned the data to the Montreal Neurological Institute (MNI) 152 standard space (2009c) template, and the data was blurred with a 4 mm full-width half-max filter and masked using *3dAutomask* algorithms. Frames were registered to the minimum outlier and then aligned to standard space. We used *anaticor* ( Jo *et al*., 2010) to regress out the white matter signal and remove the effects of motion. The final result of the AFNI processing was converted to NIFTI using AFNI *3dAFNIto NIFTI.* All scans completed AFNI processing.

The anatomical scans were defaced using *pydeface* before organizing in BIDS format. Skull-stripping and registration were performed on the undefaced anatomical scans.

All code is available on GitHub ( Day, 2019).

### Organization

Data are organized according to the Brain Imaging Data Structure (BIDS) ( Gorgolewski *et al*., 2016). All 47 subjects have two sessions, with corresponding func/ and anat/ directories.

The AFNI-processed data are included in derivatives, matching the format of Nifti/. Also included for convenience are skull-stripped anatomical images, as skull-stripping is known to occasionally fail on defaced images.

Finally, individual scans have matching JSON files in both datasets, created by *dcm2niix_afni*. Supplementing these files are higher level JSON files (following the naming convention task-ANT?_bold.json) that supply the “TaskName” and “SliceTiming” parameters. Slice timing information is required by the BIDS format, and as the pre-processed (“derivatives”) data has been slice-timing corrected, an array of zeros is provided for this field.

Task timing data are included on the scan level. The “onset” and “duration” columns are in seconds, and the “trial_type” column includes cue events (“CenterCue,” “SpatialCue,” “NoCue”), target events (“Congruent,” “Incongruent”), and cue/target errors (“CueErr,” “TargetErr”). Only correct-response trials are included. Errors are also generated when the subject responded too early or not at all.

The processing script ( afniscript.sh) and demographic information ( demographics.csv) are included at the top level.

## Data availability

### Underlying data

OpenNeuro: ANT: Healthy aging and Parkinson’s disease. https://doi.org/10.18112/openneuro.ds001907.v2.0.3 ( Day *et al*., 2019)

This project contains the following underlying data:

- sub-RC4101/ – sub-RC4227/ (scans of the 46 participants at two sessions each)

These folders each contain the following underlying data:

- ses-1/anat (T1w.json and defaced T1w.nii.gz files for session 1)
- ses-1/func (bold.json, bold.nii.gz and events.tsv files for runs 1–6 of session 1)
- ses-2/anat (T1w.json and defaced T1w.nii.gz files for session 2)
- ses-2/func (bold.json, bold.nii.gz and events.tsv files for runs 1–6 of session 2)

### Extended data

OpenNeuro: ANT: Healthy aging and Parkinson’s disease. https://doi.org/10.18112/openneuro.ds001907.v2.0.3 ( Day *et al*., 2019)

This project contains the following extended data:

- .bidsignore (file to suppress BIDS naming warning messages)
- afniscript.sh (processing script)
- dataset_description.json (BIDS dataset parameters)
- demographics.csv (demographic information for participants)
- README (README file, including changelog)
- task-ANT_bold.json (acquisition parameters for task scan)
- derivatives/ (AFNI-processed functional images within func/ directories; skull-stripped anatomical images within anat/)

Data are available under the terms of the Creative Commons Zero "No rights reserved" data waiver (CC0 1.0 Public domain dedication).

## Software availability

- Source code available from: https://github.com/IBIC/UdallANT
- Archived source code at time of publication: https://doi.org/10.5281/zenodo.2847832 ( Day, 2019)
- License: MIT
