# Supplementary material for: Attention Network Test fMRI data for participants with Parkinson’s disease and healthy elderly
Source: F1000Res. 2019 Jun 4;8:780. [Version 1] doi: 10.12688/f1000research.19288.1 (PMC7217223; doi:10.12688/f1000research.19288.1)
Supplement: Peer review history

# Peer review: Reviewer response for version 1

**DOI:** 10.5256/f1000research.21144.r62815

**Published:** 2020-05-11

**Authors:** Yong Jeong

**Affiliations:** 1 Department of Bio and Brain Engineering, Korea Advanced Institute of Science and Technology, Daejeon, South Korea

This data note is on the brain fMRI data of patients with Parkinson’s disease in Openneuro.

 They provide unprocessed and also preprocessed fMRI data from 25 patients and 21 healthy controls acquired during the attention network test tasks along with T1 structural MRI. The MRI acquisition parameters and preprocessing codes are also provided thus other researchers can replicate the results or use them for other purposes such as machine learning-based classification. They also provide the basic demographics and the performance of cognitive tests.

 Given increased activities of neuroimage data sharing, the data has values of providing unique fMRI data from patients. There are available task fMRI data from normal controls, however, limited data from patients with neurological diseases or psychiatric disorders. Furthermore, patients with Parkinson’s disease showing motor problems have difficulties in performing taskㄴ in the scanner and commonly have motion artifacts.

 There are some items needed for the wide use of the data. First, the authors need to provide the medication history of the patients, at least the levodopa dose equivalency. Since, the medication influence a lot on the motor and cognitive performance in the patients and also on brain activities, one may use them as covariates depending on their interests. Second, they need to provide the performance of ANT task corresponding task scan. One can separate sessions into correct or fail, omit, or commit. This approach is popular in the attention fMRI experiment. Third, it will be great if they can provide resting fMRI and/or DTI data. These data can be used for investigating the functional/structural connectivity or network change in the disease.

Are sufficient details of methods and materials provided to allow replication by others?

Partly

Is the rationale for creating the dataset(s) clearly described?

Yes

Are the datasets clearly presented in a useable and accessible format?

Yes

Are the protocols appropriate and is the work technically sound?

Yes

Reviewer Expertise:

Neuroimaging in neurological diseases

I confirm that I have read this submission and believe that I have an appropriate level of expertise to confirm that it is of an acceptable scientific standard.

# Peer review: Reviewer response for version 1

**DOI:** 10.5256/f1000research.21144.r49476

**Published:** 2019-06-19

**Authors:** Brian Berman

**Affiliations:** 1 Department of Neurology, University of Colorado Anschutz Medical Campus, Aurora, CO, USA

The authors have done a nice job presenting their imaging data that are being made available for public download. The article is well written and concise and provides background information necessary to enable the utilization of these data by other investigators. A sampling of imaging data provided online was looked at and appears to be appropriate and of good quality. The accompanying demographics file was reviewed and contains pertinent data. One issue I noticed on quick review is that there are MoCA and MMSE scores that exceed 30. Otherwise I have largely minor suggestions in order to improve the accessibility and utilization of these data by others:

- Please state reason for repeating scanning sessions. If time between two sessions may be relevant for analysis, then please provide timing log for each subject.
- Would include sex difference comparison between groups. They look like they may be different.
- BIDS in Materials section could be referenced as is done later under “Organization”.
- Would use “MDS-UPDRS” in Table 1 to distinguish values from the “UPDRS” scale.
- Tables should note if parentheses represent standard deviations. (Parentheses are incorrectly used to note units.)
- There were 149 volumes resulting in 357.6 sec of scanning time. Were the scans actually 6 min long? Were there any dummy scans at begging for T1 equilibration and if so have these been removed?
- Would explicitly state matrix size and thickness for EPI scans. Was there any gap?
- Under “Organization” do not need to define BIDS again. Also the sentence about “skull-stripped anatomical images” being included is confusing as all images provided are the defaced images.

Are sufficient details of methods and materials provided to allow replication by others?

Yes

Is the rationale for creating the dataset(s) clearly described?

Yes

Are the datasets clearly presented in a useable and accessible format?

Yes

Are the protocols appropriate and is the work technically sound?

Yes

Reviewer Expertise:

Parkinson's disease, neuroimaging

I confirm that I have read this submission and believe that I have an appropriate level of expertise to confirm that it is of an acceptable scientific standard.
